# An investigation of the apparent breast cancer epidemic in France: screening and incidence trends in birth cohorts

**DOI:** 10.1186/1471-2407-11-401

**Published:** 2011-09-21

**Authors:** Bernard Junod, Per-Henrik Zahl, Robert M Kaplan, Jørn Olsen, Sander Greenland

**Affiliations:** 1 FORMINDEP, Roubaix, France. Previous position: Department of Epidemiology, Ecole des Hautes Etudes en Sante Publique Rennes, France; 2 Norwegian Institute of Public Health, Oslo, Norway; 3 UCLA Schools of Public Health and Medicine, Los Angeles, USA; 4 Department of Epidemiology, UCLA School of Public Health, Los Angeles, USA; 5 Department of Statistics, UCLA College of Letters and Science, Los Angeles, USA

## Abstract

**Background:**

Official descriptive data from France showed a strong increase in breast-cancer incidence between 1980 to 2005 without a corresponding change in breast-cancer mortality. This study quantifies the part of incidence increase due to secular changes in risk factor exposure and in overdiagnosis due to organised or opportunistic screening. Overdiagnosis was defined as non progressive tumours diagnosed as cancer at histology or progressive cancer that would remain asymptomatic until time of death for another cause.

**Methods:**

Comparison between age-matched cohorts from 1980 to 2005. All women residing in France and born 1911-1915, 1926-1930 and 1941-1945 are included. Sources are official data sets and published French reports on screening by mammography, age and time specific breast-cancer incidence and mortality, hormone replacement therapy, alcohol and obesity. Outcome measures include breast-cancer incidence differences adjusted for changes in risk factor distributions between pairs of age-matched cohorts who had experienced different levels of screening intensity.

**Results:**

There was an 8-fold increase in the number of mammography machines operating in France between 1980 and 2000. Opportunistic and organised screening increased over time. In comparison to age-matched cohorts born 15 years earlier, recent cohorts had adjusted incidence proportion over 11 years that were 76% higher [95% confidence limits (CL) 67%, 85%] for women aged 50 to 64 years and 23% higher [95% CL 15%, 31%] for women aged 65 to 79 years. Given that mortality did not change correspondingly, this increase in adjusted 11 year incidence proportion was considered as an estimate of overdiagnosis.

**Conclusions:**

Breast cancer may be overdiagnosed because screening increases diagnosis of slowly progressing non-life threatening cancer and increases misdiagnosis among women without progressive cancer. We suggest that these effects could largely explain the reported "epidemic" of breast cancer in France. Better predictive classification of tumours is needed in order to avoid unnecessary cancer diagnoses and subsequent procedures.

## Background

Between 1980 and 2005, age-standardized cancer incidence in France increased by 38%, primarily due to increased reported prostate cancer incidence in men and breast and lung cancer among women [1]. The case-fatality rate of breast cancer estimated from incidence and mortality decreased from 39% in 1980 to 23% in 2005. The increase in breast cancer incidence may be related to increasing exposure to causal factors, such as use of hormone replacement therapy (HRT), alcohol, obesity and change in family size, but may also be an artefact of increased screening.

**Figure 1:**
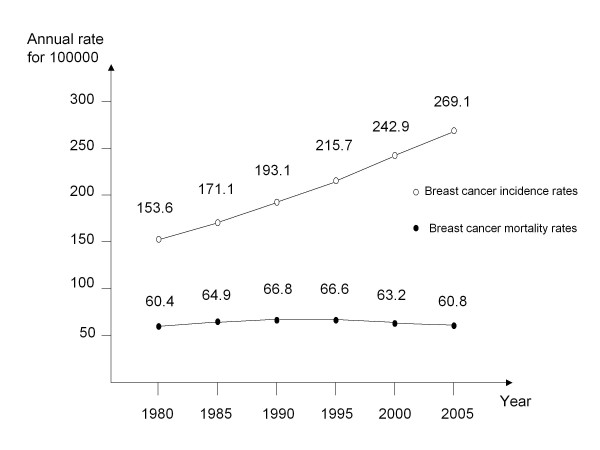
**Age standardised breast-cancer deaths and breast-cancer incidence by calendar year in France**. Standard: age structure of women aged 35 and more in 1992.

Reports from the International Agency for Research on Cancer (IARC) and from the French National Institute for Health Research (INSERM) considered the distinction between real and artificial increases in cancer frequency in France by emphasizing mortality data over incidence data [2,3]. When comparing the trends between cancer sites, the IARC report hypothesised that the net impact of early detection methods is to increase reported cancer incidence independently of environmental or lifestyle risk factors. Figure 1 shows breast cancer incidence and breast cancer mortality for the period 1980 to 2005, revealing a substantial discrepancy. If the true incidence in breast cancer was not increasing over time, both screening and improvements in treatment should have substantially reduced breast-cancer mortality.

The goal of breast-cancer screening (testing for the disease in asymptomatic patients) is to reduce mortality by diagnosing and treating tumours earlier in the disease process. Initially, screening programs will increase rates of cancer diagnosis because prevalent tumours are detected earlier. After the introduction of screening, when the reservoir of undiagnosed cases is depleted, a decline of incidence is expected before a new steady state is achieved [4]. However, recent papers suggest that publicly available mammography screening programs are associated with 10% to 50% overdiagnosis [5,6], where overdiagnosis is defined as the detection, through screening, of disease that would never have been diagnosed in the absence of screening and thus unlikely to have imposed health consequences throughout life [7]. Increase in screening activity also occurs without organized screening program. For example, after careful modelling, overdiagnosis was over 40% for the younger cohorts that had been exposed to mammograms in Catalonia [8].

A Norwegian study suggested that mammography screening leads to a larger increase in detected invasive breast cancer than can be explained by earlier diagnosis or increased exposure to risk factors. The authors suggested that mammography screening detects many tumours that otherwise would spontaneously regress [9].

Most breast cancers are diagnosed by biopsy following identification by self palpation, clinical examination by a physician, or by mammography. Overdiagnosis is inevitable when testing for asymptomatic disease in almost all screening programs. Clinicians use histology for diagnosing a true progressive disease that would metastasise and cause death without treatment if no other health problem interfered with its progression. The validity of testing for true progressive cancer by histology depends on the sensitivity and the specificity of slides from the biopsy. The number of diagnosed cancer cases in an examined population is the sum of women with progressive cancer correctly diagnosed and of women diagnosed with a cancer that would not progress to clinical detection in their lifetime. The number of true progressive cancers detected in a population reflects the frequency of examinations among women with progressive cancer, the sensitivity of diagnosis procedures before the biopsy, and the sensitivity of examination by histology.

*Global sensitivity *is the proportion of progressive cancers correctly identified in a population. All nonprogressive tumours diagnosed as cancer by histology are overdiagnoses. They reflect the frequency of examinations among women without a progressive cancer, the specificity of diagnostic procedures before the biopsy, and the proportion of women without a progressive cancer correctly identified when examined by histology. All the cancer-free women not tested contribute to increase *global specificity*: the proportion of women without a true progressive cancer correctly considered as cancer free in the population. Screening increases global sensitivity. But by doing this, it also results in decreasing global specificity, which in turn produces more overdiagnosis.

Overdiagnosis includes all nonprogressive tumours diagnosed as cancer using histology and those progressive cancers that would never cause symptoms or death during a patient's lifetime. Such cases are *functional *overdiagnoses related to a patient's outcome rather than to the physiological or structural causes of overdiagnosis. Functional overdiagnosis depends not only on the cancer but also on competing causes of death and life expectancy. It occurs more frequently when screening is performed among women with a short remaining life expectancy and when global sensitivity is high.

Our study investigates how the increase in mammography screening is associated with increase in the apparent breast-cancer incidence in France. Such information is relevant to the debate about the benefits and side effects of breast-cancer screening [10-15].

## Methods

This investigation is restricted to 1980 - 2005 and to French districts of metropolitan France, the European part of the country. Districts of metropolitan France is an English translation for "départements de la France métropolitaine".

### Data

Breast cancer deaths and the populations of women were provided by the Center for Epidemiology of Medical Causes of Death (CepiDC) [16]. The annual number of newly diagnosed invasive breast cancer, and the population of women in France were used to estimate the time trend of breast-cancer frequency. Diagnoses of invasive breast cancer were estimated from population-based cancer registries operating in France [17]. For year 1992 (the middle of the study period) the national estimate was based on 2193 reported cases [18]. Incidence of invasive cancer was provided up to 2005 by the French Institute for Public Health Surveillance [19].

Age and time specific exposure to HRT [20,21], alcohol [22], and obesity [23] in France were estimated from published data. For HRT, age specific prevalence was based on two cohorts available in France: "ESPS-EPAS" (sample from the social security registry) and "3C" (women from Bordeaux, Dijon and Montpellier). The relative risk estimates were obtained from four models used in the report [20]. Each model takes into account three types of HRT use: oestrogen only, oestrogen plus progesterone, and oestrogen plus progestin. Women were considered exposed to alcohol if they drank at least 6 glasses or more on one occasion and/or at least 14 glasses a week. Obesity was defined by a body mass index equal to or larger than 30 kg/m^2^. For alcohol and obesity, relative risk estimates were based on international literature [24].

### Changes in diagnostic procedure

We used two data sources to evaluate changes in mammography practice. First, we estimated the number of mammography machines registered annually in France, using the same methods from the 1970s to 2000 [25]. The mean number of screening tests performed per mammography machine is available for 1988 [26]. Second, we estimated the implementation of organised breast-cancer screening programs by mammography in France up to 2004 [27]. For two districts we also had the age distribution of women undergoing mammography when tested either by an organised program or by private initiative in 1995 [28].

### Change in incidence due to change in risk factor exposure

Breast-cancer incidence attributable to change in exposure to risk factors over time is obtained in age-specific categories. It is computed from incidence in the reference period, available exposure prevalence for each period, and from relative risk estimates. Additional file 1 provides formulas used for these calculations.

### Overdiagnosis estimate from change in incidence and breast-cancer mortality

Change in incidence proportion was obtained by comparing age-matched cohorts 15 years apart. Comparisons of incidence within a pair of cohorts submitted to different screening activity were performed separately for middle age women aged 50 to 64 and for elderly women aged 65 to 79. In each pair, the reference cohort was observed at an earlier calendar period when screening activity was less intense. The reference cohort was observed 15 years before the comparison cohort for both age groups of women. For middle-aged women, the reference cohort included women born between 1926 and 1930 and was compared to the cohort of women born in 1941-1945. For elderly women, the reference cohort included women born in 1911-1915 and was compared to the 1926-1930 birth cohort. For both middle age and elderly women, incidence was observed yearly for 5-years age groups. Change in crude incidence proportion associated with a 15 year change in screening activity is the difference between 11 years incidence proportion within each pair of cohorts. The detailed computation of incidence proportion is given in additional file 2. The same procedure was used for breast-cancer mortality.

Within each pair of cohorts, incidence attributable to change in risk factor prevalence and to change in mortality proportion, if any, was subtracted from crude incidence proportion to get an estimate of overdiagnosis between the two cohorts in the comparison.

### Statistical Methods

Confidence limits (CL) were obtained from a normal approximation to the distribution of proportions for the comparison of initial procedure leading to breast-cancer diagnosis over time. Confidence limits for differences between incidence proportions were obtained using French official data and observed cases in French cancer registries operating in 1992 [18]. Confidence limits were not calculated for estimates of the full population. See additional file 2 for formulas.

## Results

### Time trend for availability and use of mammography screening

The number of mammography machines increased steadily from 308 in 1980, 499 in 1984, 1351 in 1990, 2282 in 1994 to 2511 in 2000. There were about 8 times more mammography machines available in 2000 than in 1980.

There were three districts with an organised screening program in 1989, 13 in 1994 and 31 in 1999. In 2004, all 96 districts had an organised screening program. Screening began at age 50, and in 1999, the upper age limit for inviting women to be screened every second year was extended from 69 up to 74 years of age. During the whole period, screening practices were not restricted to the women included in organised programs.

In two districts with an organised program in 1995, the mammography rate before 50 or after 69 years of age was 59% of the mammography rate in the organized screening program for women aged 50 to 69 [28]. In 1988, the mean number of screening mammography per mammography machine amounted to 1050 per year [26,28].

### Time trend of exposure to risk factor

Changes in exposure to risk factors are summarised in Table 1. In comparison to 1980-1990, there was an increased prevalence of HRT use and obesity by 1995-2005, whereas alcohol consumption in women decreased.

**Table 1:** Change over time in the prevalence of risk factor exposure

Age group	**HRT (RR = 1.17 **[21]**^a^)**		**Alcohol (RR = 1.7 **[24]**^b^)**		**Obesity (RR = 2.0 **[24]**^c^)**	
	1980-1990	1995-2005	1980-1990	1995-2005	1980-1990	1995 -2005
50-59	7.9%	31.6%	16.7%	13.5%	4.1%	6.4%

60-69	7.7%	30.7%	7.0%	5.7%	6.1%	10.4%

70-79	2.3%	9.0%	3.7%	3.0%	6.1%	10.4%

^a ^RR resulting from the four available models in table six of "AFSSAPS report"[21]. RR = Total of expected exposed cases (3922.15)/Total of expected non-exposed cases (3358.78) = 1.17

Prevalence data restricted to population based samples: "ESPS-EPAS" and "3C" [21].

^b ^Interpolation between delivered results (1.5 and 2.0)

^c ^Prevalence in age group 60-69 was extrapolated to age group 70-79

### Changes over time in breast-cancer incidence and breast-cancer mortality

**Figure 2:**
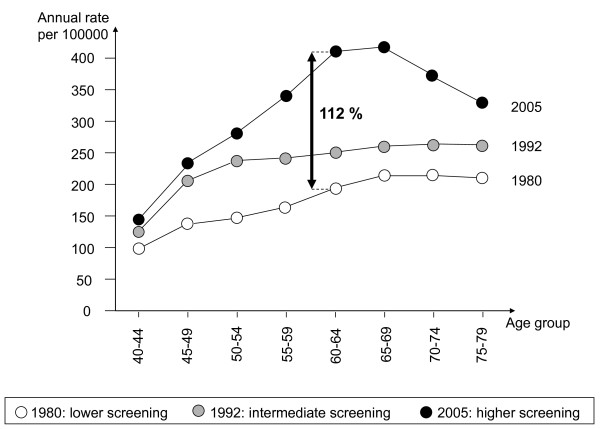
**Breast-cancer incidence rates by age and according to screening activity**. France, 1980, 1992 and 2005.

Figure 2 shows the age-specific increase of breast-cancer incidence over time. The largest increase occurred for women 45 to 74 years of age. For women aged 50 to 69, the incidence in 2005 is twice the incidence in 1980. The largest increase occurred in 2005 for the 60 to 64 age group. In 2005, the breast-cancer incidence decreased after age 74 compared to rates for women aged 60-69; the shape of the age-specific incidence rate changed from being non-declining with age to being bell shaped.

**Figure 3:**
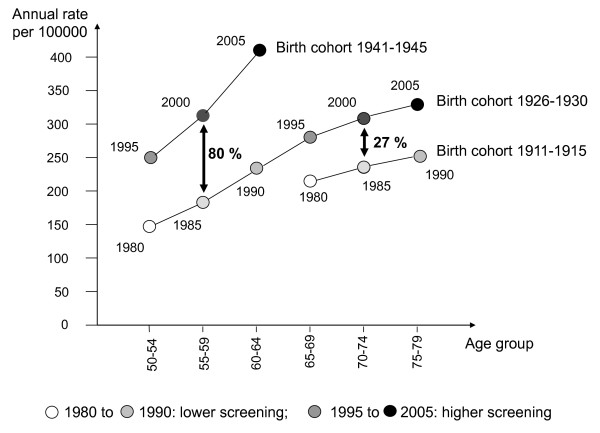
**Breast-cancer incidence in birth cohorts subject to different levels of screening activity**. Crude incidence proportion increase in %. France, 1980 to 2005.

In cohorts that had more intensive breast screening, we might expect a reduction of breast-cancer incidence after age 74 since slow growing tumours should have been detected but this was not seen. Within each pair of cohorts, the observed increase in incidence is even larger at the end of the period of comparison than 11 years before. This is visible in Figure 3 for age groups 65-69 to 75-79. Age-specific breast-cancer mortality was similar in the two pairs of cohorts. In the pair of cohorts of middle age women (50 to 64), the cumulative breast-cancer mortality rates were 6.7/1000 from 1980 to 1990 and 6.6/1000 from 1995 to 2005. In the cohorts of elderly women (65-79), the cumulative breast-cancer mortality rates were 9.9/1000 from 1980 to 1990 and 10.7/1000 from 1995 to 2005.

### Estimates of overdiagnosis

Incidence rates observed in the cohorts are given in Figure 3. For women aged 50 to 64, the 11-years incidence proportion was 20/1000 in the reference cohort observed from 1980 to 1990. It increased 80% [95% CL: 72%, 89%] to 37/1000 in the age-matched cohort observed from 1995 to 2005. For women aged 65 to 79, the 11-year incidence proportion was 24/1000 in the reference cohort observed from 1980 to 1990. It increased 27% [95%CL 20%, 34%] to 31/1000 in the age-matched cohort observed from 1995 to 2005.

Overdiagnosis estimates take into account changes in incidence proportions, and adjustments due to change in risk factors prevalence, as given in Table 2. Given that breast-cancer mortality changed less than 0.1/1000 per year, and moved in opposite direction according to age group, it was not taken into account for adjustments. Overdiagnosis estimates are slightly lower than the crude difference between incidence proportions within each pair of cohorts. Adjustment for increase in HRT use and in obesity resulted in a slight reduction in the crude difference between incidence proportions. On the contrary, decrease in alcohol consumption contributed to a small increase in the overdiagnosis estimate for each considered pair. Overdiagnosis estimates from 1995 to 2005 were 76% (95% CL: 67%; 85%) for women aged 50 to 64 and 23% [95% CL: 15%; 31%] for women aged 65 to 79.

**Table 2:** Breast-cancer incidence proportion from 1980-1990 to 1995-2005 in France

Age in the cohort	Incidence proportion of breast cancer diagnosis for 11 year periods		Incidence proportion attributable to change in risk factor exposure from 1980-1990 to 1995-2005			Relative change in adjusted incidence proportion attributable to overdiagnosis
	*Cases per 1000*		*Cases per 1000*			*Adjusted relative increase*
	*(1)*	*(2)*	*(3)*	*(4)*	*(5)*	*{(2)-(1)-(3)-(4)-(5)}/(1)*
	1980 - 1990	1995 - 2005	HRT	Alcohol	Obesity	
50-64	*20.4 *	*36.8*	*.82*	*- .40*	*.48*	*76.0%* *95% CL:66.7; 85.0*

65-79	*24.3*	*30.9*	*.89*	*- .25*	*.33*	*23.0 *% *95% CL: 15.2%; 30.9%*

Comparison of 11 year follow-up of age matched cohorts 15 years apart

## Discussion

We observed that standardised breast-cancer incidence rates increased steadily from 1980 to 2005 as use of screening tools increased, whereas age standardised breast-cancer mortality rates changed only slightly during this period. These trends might reflect a progressive increase in unknown breast cancer exposure and a decrease in case fatality due to better treatment. However, our results are consistent with other studies that fail to demonstrate a benefit of screening for breast cancer at the population level.

Opinion on the value of screening mammography remains divided. Many investigators, particularly from the radiology community, support population screening [10,12,29]. On the other hand, some but not all meta-analysis of randomised controlled trials fail to document survival benefits [14]. Meta analyses may come to different conclusions because they apply different study exclusion criteria. Those with stricter quality criteria tend to favour the null effects of screening, particularly for women younger than 50 years. However, some have argued that the choice in quality criteria is subjective [12] or due to assumptions [11]. Further, systematic quasi-experimental evaluations in Norway reported poor survival benefit in those screened [30]. Similar studies from Denmark suggest that the decline in breast cancer mortality was greater in regions without screening than it was in areas where screening was phased in earlier [31]. A recent study used WHO data to compare trends in breast cancer mortality in three pairs of European countries: Northern Ireland v. Republic of Ireland, Netherlands v. Belgium, and Sweden v. Norway. Although one member of each county pair had a more aggressive screening policy, reductions in breast cancer mortality were similar for in all three pairs. These findings are consistent with clinical trials and other quasi-experiments that have failed to show significant reductions in mortality directly attributable to mammography [32].

Well-conducted screening programs should lead to an initial increase of both prevalent cases and lead time, and then to a subsequent decline of observed advanced tumours which was not observed. After 74 years of age, when women are not invited by organised screening programs, the incidence rate should not increase as much as when screening occurred previously in the cohort [33]. Nonetheless, compared to earlier ages, the increase in breast-cancer incidence was even larger for women aged 75-79 in 1995 than in 1990, when screening was less intense up to 74 years of age. The unexpected increase in cancer incidence in older women may reflect overdiagnosis due to greater screening [34,35].

The period of observation was chosen to ensure stability in the recording systems for both death, incidence and in the nomenclatures used. Before 1978, breast-cancer incidence was not available and breast-cancer mortality trend was biased by the fact that death due to breast cancer was also declared as "cancer" without specifying the site of the primary tumour. Statistics are available on the age frequency distribution of surgical interventions performed in 1999 for breast cancer in France [36]. Among women aged 50 to 79, there are 16.5% more interventions than incident cases of invasive cancer. This difference is consistent with inclusion of women operated more than once or for ductal carcinoma in situ.

Our study has several important limitations. Only a small proportion of the French population is included in cancer registries (about 7% in the middle of the study period). In addition, we can not rule out secular changes in other breast-cancer risk factors like age at first birth, nulliparity or socioeconomic status. Adjustments for changes in HRT, alcohol and obesity prevalence over time are by nature imperfect.

Organised screening programs do not give an unbiased appraisal of actual screening activity in France: they do not include opportunistic screening, which is substantial [28]. An increase over time in the number of mammography machines in France is likely to explain changes of initial procedures leading to breast-cancer diagnosis, as shown in a study conducted in the district of Haute Vienne [37]. During 1986-1989, 80% of the cancers (298 of 372) were discovered by the patient, while this proportion fell to 52% (176 of 341) during 1997-1998. The difference between the two groups is 28.5% [95% CL 21.8%, 35.2%]. This reduction was primarily offset by the increase in the proportion of breast cancer discovered by mammography: 24.5% [95% CL 20.2%; 28.8%]. The shorter duration of the second period indicates increased frequency of breast-cancer diagnosis. This increase observed 10 years apart in the district of Haute Vienne is about 2/3 of the corresponding increase observed 15 years apart in cohorts from 50 to 79 years of age at the national level.

A 1% drop of global specificity would explain the observed increase in breast-cancer incidence in France. Suppose that among 1000 women, 4 have undetected true invasive breast cancer and 996 do not have invasive breast cancer. If the women are not examined, the four cases will eventually be diagnosed if they become symptomatic and the specificity is 100%. If these 1000 women undergo diagnostic procedures with a global sensitivity of 90% and a global specificity of 99%, we would get 90% of 4, that is 3.6 true positives; 99% of 996, that is about 986 true negatives; 10% of 4, or 0.4 false negative and 1% of 996, that is about 10 false positive. The positive predictive value among the 13.6 diagnosed "cancer" is thus about 3.6/13.6, less than 30%. This example illustrates how overdiagnosis may increase with screening even without changes in the intrinsic sensitivity and specificity of each diagnostic procedure.

Changes in the studied risk factors did not explain much of the increase in breast-cancer incidence over time. The emergence of overdiagnosis as a possible explanation of the incidence trend is related to the long period over which screening intensity has been increasing.

An analogous divergence between the trends in incidence and mortality was observed from 1927 through 1947 in Canada. Confidence in the efficacy of Halsted's radical procedure contributed to increasing early screening by breast self-examination. McKinnon hypothesised that the limitations of diagnosis confirmed by histology, which is "fraught with uncertainty", explained all or part of the apparent improvement in survival of cancers screened at an early stage during this period [38].

Undetected invasive breast cancers exist among women at the time of death due to other causes. Welch and Black used autopsy studies to estimate the size of the "reservoirs" of ductal cancer in situ [39]. These studies also revealed undetected invasive breast cancer among women who died from other causes [40-43]. Other publications report an elevated frequency of slowly or non-progressing lesions [44], some likely to be misdiagnosed as invasive cancer [45]. It is therefore possible that opportunistic screening explains most of the excess of overdiagnosis before age 50 and after age 74. Similar breast-cancer mortality in the cohort observed from 1980 to 1990 and the cohort observed from 1995 to 2005 also indicates overdiagnosis as a possible explanation for the incidence increase from age 40 to 79.

The 2003 report of the French Cancer Commission furnishes a key to interpreting this increased incidence: "Overdiagnosis (diagnosis of tumours at the borderline of malignancy) constitutes a serious problem because it can artificially increase the incidence of cancer and the result of treatment" [46]. In 2005, a discussion at the French Academy of Medicine suggested that the definition of cancer should change to include evidence of the progression of the tumour over time [47].

## Conclusion

In summary, there has been a substantial increase in breast-cancer incidence in France without a corresponding increase in mortality. Although this could be explained by a perfect balance between an increased incidence and improved survival, we think the increased breast-cancer incidence observed in France since 1980 largely reflects an increase in overdiagnosis. The latter includes misdiagnoses and true cancer lesions that would not have had any impact on the health of the women during their normal lifetime. Better predictive classification of tumours is needed in order to avoid unnecessary cancer diagnoses and subsequent procedures.

## Competing interests

The authors declare that they have no competing interests.

## Authors' contributions

BJ: Choice of the topic of the study. Data gathering, data analysis and contribution to writing. PHZ: Definition of specific research objectives, study design and contribution to writing. RMK: Elaboration of the structure of each section and contribution to writing. JO: Choice of epidemiological methods and of appropriate data. Contribution to writing. SG: Choice of statistical methods and of appropriate analysis. Contribution to writing.

All authors read and approved the final manuscript.

## Pre-publication history

The pre-publication history for this paper can be accessed here:

http://www.biomedcentral.com/1471-2407/11/401/prepub

## Supplementary Material

Additional file 1 **Formulas for change in incidence after change in exposure prevalence for hormone replacement therapy, alcohol and obesity**. Explanation of the formulas used for estimating changes in breast-cancer incidence after change in risk factor exposure. Click here for file

Additional file 2 **Formulas for incidence proportion and confidence limits in cohorts**. Explanation of the formulas used for estimating incidence proportion and confidence limits in cohorts. Click here for file
