# Supplementary material for: An investigation of the apparent breast cancer epidemic in France: screening and incidence trends in birth cohorts
Source: BMC Cancer. 2011 Sep 21;11:401. doi: 10.1186/1471-2407-11-401 (PMC3188513; doi:10.1186/1471-2407-11-401)
Supplement: Additional file 1 — Formulas for change in incidence after change in exposure prevalence for hormone replacement therapy, alcohol and obesity. Explanation of the formulas used for estimating changes in breast-cancer incidence after change in risk factor exposure. [file 1471-2407-11-401-S1.DOC]

**Formulas for change in incidence after change in exposure**

**prevalence (p’_e_ – p_e_) for hormone replacement therapy, alcohol and obesity**

For each risk factor, the change in incidence over time from *I*  to *I ’* was obtained from a model where observed incidence *I* is a weighted sum of incidence among non-exposed (*I_o_*) and exposed (*I_e_)* women , as shown in equation [a].

*I =   I_o_ • (1 - p_e_) + I_e_  • p_e_* [a]

The relative risk (*RR*) is given in equation [b]

*RR = I_e_ / I_o_* [b]

Equation [c] gives the expected incidence (*I ’* ) where the level of age- and time-specific exposure prevalence has moved from p_e_ to *p_e_’* .

*I ’  =   I_o_ • (1 – p_e_’) + I_e_  • p_e_’* [c]

The model is defined by equations [a], [b], and [c]. Unknown are I_o_, I_e_ , and I’. From these equations, the change in incidence (*I ’ - I* ) attributable to the change in risk factor prevalence will be expressed as a function of *I*, *p_e_*, *p_e_’*, and RR.

From equation [b],

I_o_ = I_e_ / RR [d]

When replacing I_o_ in equation [a] by its value obtained from equation [d],

I =    (I_e_ / RR) • (1 - p_e_) + I_e_ • p_e_ [e]

###### Hence,

###### I =    I_e_ • {1/RR - p_e_ /RR + p_e_ } [f]

Hence

I =    I_e_ • {1/RR - p_e_ • (1/RR – 1) } [g]

Similarly, from c and d,

I’ =    I_e_ • { 1/RR – p’_e_ • (1/RR – 1) } [h]

From [g],

I_e_ = I / {1/RR - p_e_ • (1/RR – 1) } [i]

hence,

I_e_ = I • RR / {1 + p_e_ (RR –1)} [j]

From [j] and [h],

I’ =    I • RR / {1 + p_e_ (RR –1)} • {1/RR – p’_e_ • (1/RR – 1) } [k]

When reordering and replacing the product RR • 1/RR by 1, it becomes

I’ =    I • {1 + p’_e_ • (RR-1) } / {1 + p_e_ (RR –1)} [l]

When substracting I to both sides of equation [l]

I’ − I = I • { {1 + p’_e_ • (RR-1) } / {1 + p_e_ (RR –1)} -1} [m]

hence,

I’ − I = I • { {1 + p’_e_ • (RR-1)} - {1 + p_e_ (RR –1)} } / {1 + p_e_ (RR –1)} [n]

hence,

I’ − I = I • {p’_e_ • (RR –1) - p_e_ (RR –1)} / {1 + p_e_ (RR –1)} [o]

I’ − I = I • (p’_e_ - p_e_ )• (RR –1) / {1 + p_e_ (RR –1)} [p]

This difference was obtained separately for HRT (I’ − I), alcohol (I’’ − I), and obesity (I’’’ − I). Assuming independence of prevalence changes, and no interaction between risk factor specific RRs, the expected change in incidence due to HRT, alcohol and obesity is then

(I’ − I ) + (I’’ − I) + (I’’’ − I) = I’ + I’’ + I’’’ − 3 • I                               [q]
