# Supplementary material for: An investigation of the apparent breast cancer epidemic in France: screening and incidence trends in birth cohorts
Source: BMC Cancer. 2011 Sep 21;11:401. doi: 10.1186/1471-2407-11-401 (PMC3188513; doi:10.1186/1471-2407-11-401)
Supplement: Additional file 2 — Formulas for incidence proportion and confidence limits in cohorts. Explanation of the formulas used for estimating incidence proportion and confidence limits in cohorts. [file 1471-2407-11-401-S2.DOC]

**Formulas for incidence proportion and confidence limits in cohorts**

A cohort is a population of women born within 5 consecutive years. Official data provide cases and rates in France for each calendar year in contiguous 5-year age groups. For each cohort, cases and populations are available every fifth year, from 1980 to 2005, without interpolation. For intermediate years, populations and cases are obtained by interpolation of available data on contiguous five year cohorts.

Cases officially provided for France result from models run on available data of French cancer registries. For computing confidence limits, we take into account the actual number of available cases in 1992, the middle of the study period.

***Populations (P), Cases (C) and Incidence Proportion (IP)***

*i:* age group varying by 5 years: 40-44, 45-49,…

*j:* calendar year

*n: j* modulo 5; *n* varies from 0 to 4 for incrementing an *i^th^* age group

*i,n:* 5 years age group shifted by *n* years. For *i =* 40-44 and *n =* 1, then *i,n =* 41-45*.* When *j* increases by 5, *i*  increases by one five year age group. Calendar time and age are tied.

*R*: rate

*P_i,n,j_ = P_i,j_ • (1 - 0.2n) + P_i+1,j+5_ • (0.2n)*

*C_i,n,j_ = C_i,j_ • (1 - 0.2n) + C_i+1,j+5_ • (0.2n)*

*R_i,n,j_ = C_i,n,j_ / P_i,n,j_*

For birth cohort *b,* from year *j= f* and for a duration of *d* years

*C_b,f,d_ = _j=f_ Σ _f+d-1_  C_i,j_ • (1 - 0.2n) + C_i+1,j+5_ • (0.2n)*

*IP_b,f,d_ = 1 – { _j=f_ Π _j=f+d-1_ [1 - R_i,n,j_] }*

***Confidence limits of the crude difference (D) between IP and IP’***

During year 1992, the middle of the study period, 2193 incident cases were provided by cancer registries. For the same year, official estimate for the total number of cases in France was 31818. The applicable ratio for estimating the variability of a number of cases is 2193/31818=.0689.

The actual number of cases following a Poisson distribution was estimated by applying the correction factor 0.0689 to *C_i,j_* and *C_i+1,j+5_* for getting respectively *CC_i,j_* and *CC_i+1,j+5_.*

The variance *V* of the adjusted sum of cases *CC_b,f,d_* obtained by interpolation is

*V (CC_b,f,d_) = _j=f_ Σ _j=f+d-1_  CC_i,j_ • (1 - 0.2n)^2^ + CC_i+1,j+5_ • (0.2n)^2^*

Let *IP_b,f,d_* = *V(CC_b,f,d_ )* */ x*

Then *V* (*IP_b,f,d_*) = *V(CC_b,f,d_) • (1/x)^2^ where (1/x) = ( IP_b,f,d_* */V( CC_b,f,d_)*

*V* (*IP_b,f,d_*) = *V(CC_b,f,d_ ) * (IP_b,f,d_ / V( CC_b,f,d_))^2^ = (IP_b,f,d_) ^2^ / V( CC_b,f,d_ )*

When comparing incidence proportions obtained in a pair of cohorts, *IP_b,f,d_* and *IP’_b’,f’,d_,* the variance of the difference *V(D)= V* *(IP_b,f,d_) + V (IP’_b’,f’,d_ ).*

The 95% confidence limits are *D – 1.96 • [ V(D)] ^.5^; D + 1.96 • [V(D)] ^.5^*

***Confidence limits of the adjusted difference (D’) between incidence proportions***

Attributable cases *(AC)* due to change in risk factor exposure between compared cohorts are obtained for each risk factor from observed incidence *I* before change in exposure and from fixed parameters (see Appendix 1). Under the assumption of a Poisson distribution of *I*, the total variance (*V_CCE_*) of the correction in incidence proportion due to change in exposure to hormone replacement therapy *(AC_HRT_),* alcohol *(AC_ALC_)* and obesity *(AC_OB_)* is estimated by:

*V_CCE_ = {AC_HRT_ + AC_ALC_+ AC_OB_} * k^2^*

where *k* is the factor used for getting attributable incidence proportion per one thousand women from *AC_HRT_ + AC_ALC_+ AC_OB_*. The variance of the adjusted difference between incidence proportions (*V(D’)*) is then :

*V(D’) = V(D) + V_CCE_*

The 95% confidence limits are: *D’ – 1.96 • [ V(D’)] ^.5^; D’ + 1.96 • [V(D’)] ^.5^*
